# Social isolation and health outcomes among older people in China

**DOI:** 10.1186/s12877-021-02681-1

**Published:** 2021-12-18

**Authors:** Yun Zhang, Wen Hu, Zhixin Feng

**Affiliations:** 1 School of Sociology & Anthropology, Sun Yat-Sen University, Guangzhou, 510275 China; 2 Zhou Enlai School of Government, Nankai University, Tianjin, 300350 China; 3 School of Geography and Planning, Sun Yat-Sen University, Guangzhou, 510275 China

**Keywords:** Social isolation, Loneliness, Cognition, Activities of daily living, Self-rated health

## Abstract

**Background:**

Social isolation is a serious public health issue affecting a significant number of older adults worldwide. However, associations between different dimensions of social isolation and functional health are unclear. We assessed the varied effects of social isolation on health among a nationwide sample of older adults from China.

**Methods:**

We assessed social isolation among 5,419 people aged 65 and older who took part in both the 2011 and 2014 waves of the Chinese Longitudinal Healthy Longevity Survey. Social isolation includes objective social isolation (kinlessness and lack of social contacts) and subjective social isolation. Four functional health outcomes were examined: self-rated health (SRH), activities of daily living (ADLs), instrumental activities of daily living (IADLs), and cognitive function measured by the Mini-Mental State Examination (MMSE). We used multivariable regression analyses to examine the associations between social isolation and health outcomes.

**Results:**

Older people who never married or who had recently lost a spouse were more likely to report poor SRH (OR=2.44) and difficulty with IADLs (ORs=1.46) than those who were married and lived with a spouse. Older people who never gave birth were less likely to report cognitive impairment (OR=0.53) than those who had living children, while older people who had recently lost a child were more likely to report poor SRH than those who had living children (OR=1.32). Older people who had no children visiting were more likely to report difficulty with IADLs than those who had children visiting (OR=1.25). In terms of subjective social isolation, older people who felt lonely were more likely to report poor SRH, cognitive impairment, and difficulty with ADLs and IADLs (ORs=1.19, 1.27, 1.28 and 1.21, respectively), and older people who had no one to talk to were more likely to report poor SRH, cognitive decline, and difficulty with ADLs and IADLs (ORs=2.08, 5.32, 2.06 and 1.98, respectively).

**Conclusions:**

Kinlessness, lack of social contacts and subjective social isolation may impact various dimensions of health in older people. Due to the varied health consequences of social isolation, targeted health interventions should be developed to address relevant situations of social isolation.

**Supplementary Information:**

The online version contains supplementary material available at 10.1186/s12877-021-02681-1.

## Introduction

Social isolation, generally defined as “the inadequate quality and quantity of social contacts” [1], is potentially a key factor when exploring the influence of the social environment on older people’s health. Social isolation may reduce social connections and diminish the sense of “coherence” or meaning and purpose in life [2], thus leading to numerous detrimental health conditions, such as depression, cardiovascular disease, and coronary heart disease or stroke [3, 4]. Older people may be at increased risk of social isolation due to declines in household size and social connectedness [5]. In addition, the rapid spread of coronavirus disease 2019 (COVID-19) has forced most countries to implement social distancing restrictions. According to the World Health Organization’s statement, older people are at the highest risk of COVID-19 [6]. Social isolation is one effect of implementing social distancing restrictions to prevent older people from being infected with COVID-19; meanwhile, the costs of social isolation cannot be ignored, as it also increases the incidence of health-related issues [7]. As the number of older people continues to increase in China, an increasing number of older people are at risk of social isolation. Therefore, social isolation among older people and its health consequences are of increasing concern.

Social isolation includes objective and subjective dimensions. Previously published studies indicated that objective social isolation, such as kinlessness (absence of a spouse, children, or siblings) and/or not participating in organizations, clubs, or religious groups [8–10], could contribute to a low quantity of social relationships [11]. Subjective social isolation reflects the negative sense of social isolation that accompanies the perception of deficiency in the desired number or quality of one’s social relationships [12]. These two aspects of social isolation may have negative impacts on health outcomes via various pathways [11]. For example, kinlessness may affect one’s health by affecting different health behaviours. Specifically, family members encourage individuals to exhibit good, healthy behaviours with respect to diet, exercise, leisure activities, and compliance with medical regimens and discourage individuals from health-damaging behaviours such as smoking and drinking [13]. Therefore, older people who do not have a spouse or children to live with may be more likely to develop unhealthy lifestyles, which might gradually result in deteriorating health status [14]. Subjective social isolation, by contrast, could be a stressor to individuals and influence their physiological processes by chronically affecting the immune, neuroendocrine, and cardiovascular systems and increasing allostatic load [15, 16]. Older people who perceive themselves to be socially isolated or lonely are more likely to develop heart disease or experience stroke [3].

This paper investigated the associations between various dimensions of social isolation and health outcomes to examine the likely mechanisms between social isolation and health outcomes and thus provide solid scientific evidence for relevant interventions and policy-making aimed at reducing the negative impacts on older people who are socially isolated.

## Data and Methods

### Data and Sampling

This study used the dataset from the 2011–2014 Chinese Longitudinal Healthy Longevity Survey (CLHLS). The CLHLS is a longitudinal survey of a nationally representative sample of Chinese people aged 65 and older using internationally compatible questionnaires [17]. It covered 23 out of 31 provinces in China, accounting for 85% of the total population in China. It collected extensive data on a large population of the oldest old individuals aged 80-112 and comparatively younger elderly individuals aged 65-79. There were 9,765 respondents in 2011, while 2,879 (29%) died before the 2014 survey, thus a total of 6,886 respondents were included in two waves. Subsequently, 820 (12%) of the 6,886 respondents were lost to follow-up in 2014, and 647 (9%) of the 6,886 respondents were excluded due to a lack of responses regarding health outcomes or key independent information. The final sample of this study comprised 5,419 respondents with valid information.

### Dependent Variables

Self-rated health (SRH), cognitive health, activities of daily living (ADLs), and instrumental activities of daily living (IADLs) were considered in this study (see Table 1). SRH was assessed by a comprehensive evaluation of the overall health status of older people. There were five categories: very good, good, fair, poor, and very poor. The categories were recoded into a binary variable: 0 for very good and good and 1 for very poor, poor and fair (i.e., [18]). Cognitive health was measured by the Chinese version of the Mini-Mental State Examination (MMSE), in which four aspects of mental functioning were tested: orientation, calculation, recall, and language. The maximum score for the MMSE was 30; lower scores indicated more severe cognitive problems. According to previous studies, a score of 18 was used as the cut-off point [19, 20]; respondents with MMSE scores of less than 18 were defined as having “cognitive impairment” in this study (coded as 1), and the rest were defined as having “no cognitive impairment” (coded as 0). ADLs and IADLs were used to assess functional health status. Difficulty with ADLs is commonly used to gauge older people’s daily performance in six basic activities: bathing, dressing, eating, using the toilet, free movement, and controlling urine and defecation. For comparability with previous studies (i.e., [14, 21]), a binary variable was constructed for ADLs, with 1 representing difficulty with any of the six ADLs and 0 representing no difficulty with any of the six ADLs. IADLs were assessed in terms of eight activities: visiting neighbours, going shopping, making food, washing clothes, walking one kilometre, carrying a 5 kg weight, crouching and standing three times, and taking public transportation. Similarly, a binary variable was constructed for IADLs, with 1 representing having difficulty with any of the eight IADLs and 0 representing having no difficulty with any of the eight IADLs. All health outcomes were measured in the 2014 dataset.

**Table 1:** Summary of Variables

Variable	Description	Coding	Percentage/Mean (N=5,419)
***Dependent variable (Health outcomes in 2014)***			
Self-reported health (SRH)	It originally contains 5 ordered scales: very good, good, fair, poor, and very poor.	0= very good and good	38.87%
		1= very poor, poor and fair	61.13%
Cognitive impairment	It is measured with the Chinese version of the Mini-Mental State Examination (MMSE); the total score range is 0–30.	0= MMSE score less than 18	79.27%
		1= MMSE more than 18	20.73%
Difficulty with ADLs	It includes 6 indexes: bathing, dressing, using the toilet, indoor walking, eating, and getting up and down.	0= no difficulty with any of the six ADLs	73.44%
		1= difficulty with any of the six ADLs	26.56%
Difficulty with IADLs	It includes 8 indexes: visiting neighbours, going shopping, making food, washing clothes, walking one kilometre, carrying 5kg weight, crouching and standing three times, and taking public transportation.	0= no difficulty with any of the eight IADLs	34.71%
		1= difficulty with any of the eight IADLs	65.29%
***Independent variable***			
***Kinlessness***			
Have no spouse	0=Married and live with spouse		38.44%
	1=Never married		1.14%
	2=Recent loss of spouse		6.24%
	3=Distant loss of spouse		54.18%
Have no children	0=Have living children		85.13%
	1=Never gave birth		2.18%
	2=Recent loss of children		10.96%
	3=Distant loss of children		1.73%
Have no siblings	0=Have living siblings		54.71%
	1=Have no siblings in lifetime		5.50%
	2=Recent loss of siblings		9.19%
	3=Distant loss of siblings		30.60%
***Lack of social contacts***			
Live alone	Those who became solitary living between 2011 and 2014	0= no	97.45%
		1= yes	2.55%
Had no children visiting	Those whose children were alive but ceased frequent visiting between 2011 and 2014	0= no	85.17%
		1= yes	14.83%
Had no siblings visiting	Those whose siblings were alive but ceased frequent visiting between 2011 and 2014	0= no	81.15%
		1= yes	18.85%
Not participating in social activities	Those who withdrew from social activities between 2011 and 2014	0= no	87.52%
		1= yes	12.48%
***Subjective social isolation***			
Felt lonely	Those who reported loneliness between 2011 and 2014	0= no	79.9%
		1= yes	20.1%
Had no one to talk	Those who were not able to find anyone to talk when in need between 2011 and 2014	0= no	95.79%
		1= yes	4.21%
Had no one to seek for help	Those who were not able to find anyone to seek for help when in need between 2011 and 2014	0= no	89.5%
		1= yes	10.5%
***Covariates (in 2011)***			
Age		Min=65 Max=114	Mean=82.08
Sex		0= female	53.37%
		1= male	46.63%
Self-rated SES	Self-rated socioeconomic status	1= lower	16.08%
		2= fair	65.12%
		3= higher	18.8%
Education	Years of schooling	Min=0 Max=20	Mean=2.64
Residence	Living in the rural area	0= no	56.74%
		1= yes	43.26%
Smoking	Smoke at present	0= no	87.61%
		1= yes	12.39%
Drinking	Drink at present	0= no	82.86%
		1= yes	17.14%
Leisure activities	It includes 3 indexes: gardening, reading, and watching TV. The score range of each item is from 0–4, presenting the frequency of doing the leisure activities: never, sometimes, monthly, weekly, every day.	Min=0 Max=12	Mean=4.64
Physical exercise	Regular exercise at present	0= no	82.89%
		1= yes	17.11%
Social services	Whether the respondent receive any social services in their community: personal care, home visit, psychological consulting, daily shopping, social and recreation, legal aid, healthcare education.	0= no	46.9%
		1= yes	53.1%

### Independent Variables

According to the definition of social isolation, there are two dimensions of social isolation: objective social isolation and subjective social isolation [22, 23]. Objective social isolation includes kinlessness and a lack of social contacts.

Regarding kinlessness, spouses, children and siblings are crucial members and networks within a family; thus, having no spouse, having no children, and having no siblings were the most influential external conditions leading to social isolation among older people [8–10]. Older people who never married, gave birth or had siblings in their lifetime developed different social networks early in their life. On the other hand, older people who recently experienced a loss of family members may have difficulties adapting to changes in their daily lives and may also suffer from negative impacts of bereavement. This could lead to social isolation, which could have negative impacts on respondents' psychological wellbeing and health. Therefore, we identified four statuses for each kinless variable: spouse situation: “0 for married and live with spouse, 1 for never married, 2 for recent loss of spouse (after 2011), 3 for distant loss of spouse (before 2011)”; children situation: “0 for have children alive, 1 for never gave birth, 2 for recent loss of children (after 2011), 3 for distant loss of children (before 2011)”; and sibling situation: “0 for have siblings alive, 1 for have no siblings in lifetime, 2 for recent loss of siblings (after 2011), 3 for distant loss of siblings (before 2011)”.

Regarding lack of social contacts, there are four variables that reflect social contacts in the CLHLS dataset. Whether individuals lived alone, whether they contacted family members, and whether they participated in social activities were considered. All were coded as a binary variable: 1 for “live alone”, “had no children visiting”, “had no siblings visiting”, and “did not frequently participate in social activities” and 0 for “did not live alone”, “had at least 1 child frequently visiting”, “at least 1 sibling frequently visiting”, and “participated in social activities frequently”.

In terms of subjective social isolation, feeling lonely, having no one to talk, and having no one to seek help from were considered. Feeling lonely was measured by the question “do you often feel lonely and isolated”, and the answers of respondents were “always”, “often”, “sometimes”, “seldom”, or “never”. We recoded these five categories into a binary variable, with 0 (not lonely) for seldom, never, and sometimes and 1 (feel lonely) for always and often. Had no one to talk and had no one to seek help from were both binary variables: 1 represented have no one to talk/seek help from when in need, while 0 represented have someone to talk/seek help from when in need.

### Covariates

Although the focus of this paper was on the effects of social isolation on health, it was important to control for other factors so that the results would be reliable. Demographic, socioeconomic, and lifestyle characteristics were obtained, including age, sex, self-reported socioeconomic status (SES), years of education, urban/rural residence, smoking, drinking, frequency of leisure activities, physical exercise, and availability of social services, all of which were used as control variables in accordance with previous reports [17]. All of the above covariates were measured in 2011 (see Table 1). Health status in 2011 was also controlled for in each model. Specifically, 2011 SRH was included in model 1, 2011 MMSE was included in model 2, 2011 ADLs of 2011 were included in model 3, and 2011 IADLs were included in model 4.

Age was calculated from the interview dates and self-reported birth date, which was verified by family members, genealogical records, ID cards, and household registrations. Self-reported socioeconomic status was categorized into three levels (favourable, intermediate, and unfavourable). Smoking at present, alcohol drinking at present and performing physical exercise regularly were dichotomized as yes vs. no. Leisure activities were calculated by summing the points for 3 activities: gardening, reading and watching TV. The availability of social services was dichotomized as yes (the respondents enjoyed any social services in their community, including personal care, home visits, psychological consultations, daily shopping, social and recreation, legal aid, or healthcare education) vs. no.

### Method

Logistic regression models were applied to analyse the associations between kinlessness, lack of social contacts, subjective social isolation and four health outcomes: poor SRH (binary variable: 1= yes, 0=no), cognitive decline (binary variable: 1= yes, 0=no), difficulty with ADLs (binary variable: 1=yes, 0=no), and difficulty with IADLs (binary variable: 1= yes, 0=no).

To investigate the impacts of social isolation on health outcomes, we examined those who were not considered to be socially isolated in 2011 but were considered to be socially isolated in 2014; thus, we were able to examine the associations of recent experiences of social isolation and health outcomes. Specifically, we examined those who did not lack social contacts (did not live alone, had at least one child visiting, and had at least one sibling visit) at T1 (year 2011) but had a lack of social contacts (lived alone, had no children visiting, and had no siblings visiting) at T2 (year 2014) and those who did not report subjective social isolation (did not feel lonely, have someone to talk, and have someone to seek help from) at T1 (year 2011) but reported subjective social isolation (felt lonely, had no one to talk, and had no one to seek help from) at T2 (year 2014). This strategy allowed us to focus on the experience of becoming socially isolated; thus, the results provide implications for those who experienced social isolation during the COVID-19 pandemic.

In addition, we conducted sensitivity analyses by applying multiple imputation for missing values and those lost to follow-up for variables of social contacts, subjective social isolation, and health outcomes. Following the common practice in the literature [24, 25], we assumed that the respondents with missing values would have the same values as those without missing if the former group had the same conditions of all covariates as the latter group. Using a similar approach of multiple imputation, health outcomes in 2014 for those lost to follow-up were also imputed. Multiple imputation is a general approach to address the problem of missing data, which aims to allow for the uncertainty about the missing data by creating several different plausible imputed data sets and appropriately combing results obtained from each of them [26]. In the sensitivity analyses of multiple imputation, the associations between social isolation and health outcomes were generally consistent with the main findings (Table S2) after we imputed missing values and missing data for those lost to follow-up (Table S3).

## Results

### Descriptive Statistics

The detailed descriptive statistics for all variables in this paper are summarized in Table 1. In terms of health outcomes, the proportions of participants who reported poor SRH, cognitive impairment, difficulty with ADLs, and difficulty with IADLs in 2014 were 61.13%, 20.73%, 26.56%, and 65.29%, respectively.

In terms of kinlessness, the proportions of participants who never married, did not give birth, and had no siblings in their lifetime were 1.14%, 2.18%, and 5.5%, respectively; the proportions of participants who lost their spouse, child, or siblings after 2011 were 6.24%, 10.96, and 9.19, respectively; and the proportions of participants who had lost their spouse, child, or siblings before 2011 were 54.18%, 1.73%, and 30.6%, respectively.

Regarding social contacts, during 2011-2014, the proportions of participants who began living alone, whose children ceased frequent visits, whose siblings ceased frequent visits, and those who withdrew from social activities were 2.55%, 14.83%, 18.85%, and 12.48%, respectively.

In terms of subjective social isolation, during 2011-2014, the proportions of participants who newly reported the experience of loneliness, no one to talk, and no one to ask for help when in need were 20.1%, 4.21%, and 10.5%, respectively.

### Multivariate Results

Table 2 presents the results from logistic regression models of associations between social isolation and health outcomes considering all the covariates of demographic, socioeconomic, lifestyle, and baseline health status factors. Regarding the associations between kinlessness and health outcomes, older people who were never married were more likely to report poor SRH (OR=2.44) than those married and living with spouses. Older people who experienced a recent loss of spouse were more likely to report difficulty with IADLs (OR=1.46), while distant loss of a spouse was not statistically significantly associated with any of the health outcomes. On the other hand, older people who did not give birth were less likely to report cognitive impairment than those who had living children (OR=0.53), while recent loss of children was associated with difficulty with IADLs (OR=1.32), and distant loss of children was not statistically significantly associated with any of the health outcomes. In addition, having no siblings (including having no siblings throughout their lifetime, recent loss of siblings, or distant loss of siblings) was not statistically significantly associated with any of the health outcomes.

**Table 2:** Associations between social isolation on SRH, cognitive impairment, difficulty with ADLs, difficulty with IADLs

	Poor SRH	Cognitive impairment	Difficulty with ADLs	Difficulty with IADLs
	Model 1	Model 2	Model 3	Model 4
	ORs (95%CI)	ORs (95%CI)	ORs (95%CI)	ORs (95%CI)
***Kinlessness***				
Have no spouse (ref.=married and live with spouse)				
*Never married*	2.44*	2.06	0.60	1.15
	(1.20,4.93)	(0.83,5.07)	(0.24,1.51)	(0.52,2.54)
*Recent loss of spouse*	1.14	0.98	1.22	1.46*
	(0.88,1.47)	(0.66,1.44)	(0.88,1.70)	(1.08,1.98)
*Distant loss of spouse*	0.92	1.11	0.88	1.08
	(0.80,1.07)	(0.88,1.38)	(0.73,1.07)	(0.91,1.27)
Have no children (ref.=have living children )				
*Did not give birth*	0.87	0.53*	1.21	0.85
	(0.56,1.34)	(0.29,0.99)	(0.71,2.06)	(0.49,1.47)
*Recent loss of children*	0.98	0.97	0.95	1.32*
	(0.81,1.18)	(0.76,1.24)	(0.75,1.19)	(1.04,1.68)
*Distant loss of children*	0.92	0.66	0.98	2.11
	(0.57,1.50)	(0.37,1.17)	(0.56,1.72)	(0.98,4.55)
Have no siblings (ref.=have living siblings)				
*Have no siblings in lifetime*	1.22	0.99	0.99	1.19
	(0.94,1.59)	(0.68,1.43)	(0.71,1.38)	(0.87,1.64)
*Recent loss of siblings*	1.20	1.00	0.89	1.02
	(0.98,1.47)	(0.72,1.39)	(0.67,1.18)	(0.81,1.28)
*Distant loss of siblings*	1.00	1.08	0.93	1.12
	(0.86,1.16)	(0.88,1.31)	(0.78,1.12)	(0.93,1.34)
***Lack of social contacts***				
Live alone	1.03	0.71	0.39**	0.65
	(0.70,1.51)	(0.40,1.26)	(0.21,0.72)	(0.42,1.02)
Had no children visiting	1.09	1.17	1.21	1.25*
	(0.92,1.30)	(0.93,1.48)	(0.98,1.49)	(1.02,1.54)
Had no siblings visiting	0.97	0.90	0.85	1.15
	(0.83,1.15)	(0.69,1.18)	(0.67,1.07)	(0.95,1.38)
Not participating in social activities	0.95	1.07	1.00	0.89
	(0.79,1.14)	(0.80,1.44)	(0.78,1.29)	(0.72,1.11)
***Subjective social isolation***				
Felt lonely	1.19*	1.27*	1.28**	1.21*
	(1.03,1.37)	(1.03,1.57)	(1.07,1.54)	(1.02,1.44)
Had no one to talk	2.08***	5.32***	2.06***	1.98**
	(1.51,2.88)	(3.75,7.55)	(1.49,2.84)	(1.30,3.01)
Had no one to seek for help	1.01	1.27	1.10	0.88
	(0.82,1.24)	(0.97,1.66)	(0.86,1.42)	(0.69,1.13)

Notes: All models adjusted for age, sex, self-rated SES, education, rural area, smoking, alcohol drinking, physical exercise, leisure activities and social services. In terms of baseline (measured in 2011) health status, Model 1adjusted for SRH in 2011, Model 2 adjusted for MMSE in 2011, Model 3 adjusted for ADLs in 2011, and Model 4 adjusted for IADLs in 2011.

* *P* <0.05, ** *P* <0.01, *** *P* <0.001

In terms of the associations between lack of social contacts and health outcomes, older people who were living alone were less likely to report difficulty with ADLs than those who lived with others (OR=0.39). Older people who had no children visiting were more likely to report difficulty with IADLs than those who had children visiting (OR=1.25). No significant associations were found between no sibling visits and any health outcomes. Not participating in social activities was not associated with any health outcomes among the participants.

Regarding the associations between subjective social isolation and health outcomes, older people who felt lonely were more likely to have deteriorated health outcomes for SRH, MMSE score, ADLs, and IADLs (ORs= 1.19, 1.27, 1.28, and 1.21, respectively). Meanwhile, older people who had no one to talk were significantly associated with higher odds of deteriorated health outcomes, including SRH, MMSE score, ADLs, and IADLs, than those who had someone to talk (ORs= 2.08, 5.32, 2.06, and 1.98, respectively). No significant associations were found between older people who had no one to seek help from and deteriorated outcomes for SRH, MMSE score, ADLs and IADLs.

## Discussion

This study investigates the associations between social isolation and health outcomes among older people in China. The results show that kinlessness, lack of social contacts and subjective social isolation can negatively impact older people’s health in different ways.

First, having no spouse or children was significantly associated with health outcomes, while having no siblings and health outcomes were not significantly associated. Actually, spouses and children are closer family members than siblings and are more likely to provide daily care for older people. Thus, older people who had experienced a recent loss of spouse or children were more likely to report difficulty with IADLs. This is due not only to the negative effect of bereavement but to changes in daily routines and behaviours that may result in a deteriorated health status [14]. On the other hand, the timing or length of time since losing a spouse or children was different. Recent losses of spouses and children have significant negative impacts on older people’s functional health in terms of IADLs, but the impacts of distant losses on older people’s health outcomes were not significant. In addition, although recent loss of children was associated with deteriorated health outcomes, older people who did not give birth were less likely to report cognitive impairment. Previous studies have reported similar results and suggested that people who never gave birth did not expect to receive social support from their children; as a result, they may be more likely to seek better income support, health care and social services than parents [14, 27]. In contrast, older people who were never married may have been from poor and vulnerable families, which may have prohibited them from getting married early; thus, with accumulation of the negative effects of vulnerability, they would likely also have poor SRH in later life.

In terms of lack of social contacts, older people who began living alone were less likely to report difficulty with ADLs. This may reflect them having to cope with daily living, which may help prevent them from developing functional limitations. Nevertheless, this result may also suggest that living alone does not actually lead to poor SRH, cognitive impairment, or difficulties with ADLs or IADLs. With the improvement of health status and economic status among older people, an increasing proportion and number of older people in China are choosing to live alone. Although older people who live alone are healthier and require fewer nursing services, they should also be encouraged to maintain contacts with family members and friends to prevent the situation of inadequate social relationships. In contrast, older people who had no children visiting were more likely to report difficulty with IADLs. This suggests that without children visiting, older people experience a reduced willingness to perform more complicated daily living activities, such as going shopping and visiting neighbours.

Regarding subjective social isolation, older people who felt lonely and had no one to talk to were more likely to report poor SRH, cognitive impairment, and difficulties with ADLs and IADLs than those who did not feel lonely or who had someone to talk to. It has been suggested that subjective social isolation could impact older people’s health through a psychological pathway, by disrupting their sense of belonging and social connection; therefore, subjective social isolation can not only affect one’s mental health but also act as a pivotal mechanism that affects other mechanisms that shape one’s physical health [28]. Our results thus reinforced this argument and further indicated that such associations may appear particularly in older people who feel lonely and have no one to talk to. Along with previously published studies [11], our study indicated that subjective social isolation was associated with poor SRH, cognitive decline, and difficulties with ADLs and IADLs in older people, even after adjusting for objective social isolation and health behaviours, which suggests that subjective social isolation may impact one’s health more comprehensively than objective social isolation among older people.

In summary, recent loss rather than distant loss of a spouse or children was negatively associated with health outcomes in older people, manifesting as a reduced ability to perform complicated daily living activities and poor SRH. Never married marital status was associated with poor SRH, while never gave birth was associated with better cognitive health. Living alone did not lead to deteriorated health, but having no children visiting was associated with a reduced ability to perform complicated daily living activities. Feeling lonely and having no one to talk to are crucial factors of subjective social isolation that are associated not only with older people’s difficulty with basic and complicated daily living activities but also with cognitive impairment and poor SRH.

### Conclusion

Kinlessness, lack of social contacts and subjective social isolation may have impacts on various dimensions of health in older people. Due to the varied health consequences, targeted health interventions plans should be developed to address relevant situations of social isolation.

### Limitations

This study also has some limitations. First, loneliness is measured in a single question on the CLHLS survey. As many previous studies assessed loneliness with 20 questions using the UCLA Loneliness Scale, a comparison of the results between this study and previous studies was not feasible. Notwithstanding, to strengthen the validity of the results for subjective social isolation, we also included the following two indicators: “had no one to talk to” and “had no one to seek help from”. Second, there were 1,467 (21%) of the 6,886 respondents were excluded due to the lost to follow-up or missing values. It should be concern that the relatively large number of drop-out participants may bias the results of the present study. However, we conducted the sensitivity analysis by imputing missing values and those lost to follow-up and the results were generally consistent with the main findings (see supplementary document). Therefore, such exclusion of respondents would be accepted as it did not bias the results.

Future research on these topics should focus on interventions for older people who are or have been socially isolated. Health interventions focused on how to support older people experiencing social isolation, particularly those experiencing feelings of loneliness or loss of a spouse, child, or siblings due to COVID-19, are increasingly needed. Prospective studies can assess the effects of interventions to prevent the adverse effects of social isolation on older people’s health outcomes.

## Supplementary Information

**Additional file 1.**
