# Supplementary material for: Social isolation and health outcomes among older people in China
Source: BMC Geriatr. 2021 Dec 18;21:721. doi: 10.1186/s12877-021-02681-1 (PMC8683828; doi:10.1186/s12877-021-02681-1)
Supplement: Supplementary file 1 — Additional file 1. [file 12877_2021_2681_MOESM1_ESM.docx]

**Supplementary Files**

**Social Isolation and Health Outcomes Among Older People in China**

Yun Zhang, Wen Hu, Zhixin Feng (CA)

**Contents**

Table S1…………………………………………………………….………………………………………Page 2

Appended demonstration of table2 with the results of covariates

Table S2…………………………………………………………….………………………………………Page 4

Variables and numbers of missing values and lost to follow-ups imputed by multiple imputation

Table S3…………………………………………………………….………………………………………Page 5

Results applied with multiple imputation for missing values and lost to follow-ups

Table S1.Appended demonstration of table2 with the results of covariate（N=5,419）

|  | **Poor SRH** | **Cognitive impairment** | **Difficulty with ADLs** | **Difficulty with IADLs** |
| --- | --- | --- | --- | --- |
|  | Model 1 | Model 2 | Model 3 | Model 4 |
|  | ORs (95%CI) | ORs (95%CI) | ORs (95%CI) | ORs (95%CI) |
| ***Kinlessness*** |  |  |  |  |
| Have no spouse (ref.=married and live with spouse) |  |  |  |  |
| *Never married* | 2.44* | 2.06 | 0.60 | 1.15 |
|  | (1.20,4.93) | (0.83,5.07) | (0.24,1.51) | (0.52,2.54) |
| *Recent loss of spouse* | 1.14 | 0.98 | 1.22 | 1.46* |
|  | (0.88,1.47) | (0.66,1.44) | (0.88,1.70) | (1.08,1.98) |
| *Distant loss of spouse* | 0.92 | 1.11 | 0.88 | 1.08 |
|  | (0.80,1.07) | (0.88,1.38) | (0.73,1.07) | (0.91,1.27) |
| Have no children (ref.=have children alive) |  |  |  |  |
| *Did not give birth* | 0.87 | 0.53* | 1.21 | 0.85 |
|  | (0.56,1.34) | (0.29,0.99) | (0.71,2.06) | (0.49,1.47) |
| *Recent loss of children* | 0.98 | 0.97 | 0.95 | 1.32* |
|  | (0.81,1.18) | (0.76,1.24) | (0.75,1.19) | (1.04,1.68) |
| *Distant loss of children* | 0.92 | 0.66 | 0.98 | 2.11 |
|  | (0.57,1.50) | (0.37,1.17) | (0.56,1.72) | (0.98,4.55) |
| Have no siblings (ref.=have siblings alive) |  |  |  |  |
| *Have no siblings in lifetime* | 1.22 | 0.99 | 0.99 | 1.19 |
|  | (0.94,1.59) | (0.68,1.43) | (0.71,1.38) | (0.87,1.64) |
| *Recent loss of siblings* | 1.20 | 1.00 | 0.89 | 1.02 |
|  | (0.98,1.47) | (0.72,1.39) | (0.67,1.18) | (0.81,1.28) |
| *Distant loss of siblings* | 1.00 | 1.08 | 0.93 | 1.12 |
|  | (0.86,1.16) | (0.88,1.31) | (0.78,1.12) | (0.93,1.34) |
| ***Lack of social contacts*** |  |  |  |  |
| Live alone | 1.03 | 0.71 | 0.39** | 0.65 |
|  | (0.70,1.51) | (0.40,1.26) | (0.21,0.72) | (0.42,1.02) |
| Had no children visiting | 1.09 | 1.17 | 1.21 | 1.25* |
|  | (0.92,1.30) | (0.93,1.48) | (0.98,1.49) | (1.02,1.54) |
| Had no siblings visiting | 0.97 | 0.90 | 0.85 | 1.15 |
|  | (0.83,1.15) | (0.69,1.18) | (0.67,1.07) | (0.95,1.38) |
| Not participating in social activities | 0.95 | 1.07 | 1.00 | 0.89 |
|  | (0.79,1.14) | (0.80,1.44) | (0.78,1.29) | (0.72,1.11) |
| ***Subjective social isolation*** |  |  |  |  |
| Felt lonely | 1.19* | 1.27* | 1.28** | 1.21* |
|  | (1.03,1.37) | (1.03,1.57) | (1.07,1.54) | (1.02,1.44) |
| Had no one to talk | 2.08*** | 5.32*** | 2.06*** | 1.98** |
|  | (1.51,2.88) | (3.75,7.55) | (1.49,2.84) | (1.30,3.01) |
| Had no one to seek for help | 1.01 | 1.27 | 1.10 | 0.88 |
|  | (0.82,1.24) | (0.97,1.66) | (0.86,1.42) | (0.69,1.13) |
| ***Baseline health status***  ***(all measured in 2011)*** |  |  |  |  |
| Poor SRH | 2.44*** |  |  |  |
|  | (2.17,2.74) |  |  |  |
| Cognitive impairment |  | 5.92*** |  |  |
|  |  | (4.80,7.30) |  |  |
| Difficulty with ADLs |  |  | 7.22*** |  |
|  |  |  | (6.00,8.70) |  |
| Difficulty with IADLs |  |  |  | 3.90*** |
|  |  |  |  | (3.36,4.52) |
| ***Covariates (all measured in 2011)*** |  |  |  |  |
| Age | 1.01*** | 1.10*** | 1.08*** | 1.11*** |
|  | (1.01,1.02) | (1.08,1.11) | (1.07,1.09) | (1.10,1.12) |
| Male | 0.96 | 0.77** | 0.93 | 0.58*** |
|  | (0.84,1.09) | (0.63,0.93) | (0.78,1.10) | (0.50,0.69) |
| SES | 0.70*** | 0.82** | 1.17* | 0.93 |
|  | (0.63,0.78) | (0.71,0.95) | (1.03,1.33) | (0.82,1.06) |
| Social services | 1.05 | 1.07 | 1.04 | 1.34*** |
|  | (0.93,1.18) | (0.91,1.27) | (0.90,1.21) | (1.16,1.54) |
| Education | 1.01 | 1.00 | 1.01 | 1.00 |
|  | (0.99,1.02) | (0.98,1.02) | (0.99,1.03) | (0.98,1.02) |
| Rural | 0.96 | 0.73*** | 0.70*** | 0.83** |
|  | (0.85,1.08) | (0.62,0.87) | (0.60,0.82) | (0.71,0.96) |
| Smoke | 0.91 | 0.81 | 1.05 | 0.97 |
|  | (0.76,1.09) | (0.62,1.07) | (0.82,1.35) | (0.78,1.20) |
| Drink | 1.34*** | 1.31* | 1.24* | 1.24* |
|  | (1.15,1.56) | (1.02,1.68) | (1.00,1.54) | (1.04,1.50) |
| Leisure | 0.96*** | 0.89*** | 0.95*** | 0.95*** |
|  | (0.94,0.98) | (0.86,0.92) | (0.93,0.98) | (0.93,0.98) |
| Physical exercise | 0.98 | 0.86 | 1.03 | 0.83 |
|  | (0.83,1.16) | (0.66,1.12) | (0.83,1.28) | (0.68,1.01) |

* *P* <0.05, ** *P* <0.01, *** *P* <0.001

Table S2. Variables and numbers of missing values and lost-to-follow-ups imputed by multiple imputation

| Variables | Complete | Incomplete | Imputed | Total |
| --- | --- | --- | --- | --- |
| Poor SRH | 5554 | 1332 | 1332 | 6886 |
| Cognitive impairment | 6067 | 819 | 819 | 6886 |
| Difficulty with ADLs | 5707 | 1179 | 1179 | 6886 |
| Difficulty with IADLs | 5891 | 995 | 995 | 6886 |
| Had no children visiting recently | 5419 | 1447 | 1447 | 6886 |
| Had no siblings visiting recently | 5812 | 1074 | 1074 | 6886 |
| Feel lonely recently | 6619 | 267 | 267 | 6886 |
| Had no one to talk recently | 5827 | 1059 | 1059 | 6886 |
| Had no one to seek for help recently | 5419 | 1447 | 1447 | 6886 |

Table S3. Results applied with multiple imputation for missing values and lost-to-follow-ups（N=6,886）

|  | **Poor SRH** | **Cognitive impairment** | **Difficulty with ADLs** | **Difficulty with IADLs** |
| --- | --- | --- | --- | --- |
|  | Model S1 | Model S2 | Model S3 | Model S4 |
| ***Kinlessness*** |  | | | |
| Have no spouse (ref.=married and live with spouse) |  | | | |
| *Never married* | 1.48 | 1.10 | 0.52 | 1.16 |
|  | (0.60,3.64) | (0.30,4.08) | (0.17,1.60) | (0.43,3.13) |
| *Recent loss of spouse* | 1.10 | 0.92 | 1.18 | 1.50** |
|  | (0.85,1.42) | (0.62,1.35) | (0.87,1.60) | (1.12,2.20) |
| *Distant loss of spouse* | 0.92 | 1.05 | 0.90 | 1.14 |
|  | (0.79,1.06) | (0.84,1.32) | (0.75,1.08) | (0.97,1.34) |
| Have no children (ref.=have children alive) |  |  |  |  |
| *Did not give birth* | 1.09 | 0.76 | 1.50 | 0.88 |
|  | (0.67,1.77) | (0.40,1.44) | (0.88,2.56) | (0.48,1.60) |
| *Recent loss of children* | 0.97 | 1.04 | 1.02 | 1.30* |
|  | (0.80,1.18) | (0.81,1.34) | (0.81,1.28) | (1.03,1.66) |
| *Distant loss of children* | 0.92 | 1.15 | 1.02 | 0.96 |
|  | (0.79,1.07) | (0.95,1.39) | (0.86,1.22) | (0.79,1.15) |
| Have no siblings (ref.=have siblings alive) |  |  |  |  |
| *Have no siblings in lifetime* | 1.20 | 1.17 | 1.12 | 1.18 |
|  | (0.90,1.61) | (0.74,1.83) | (0.78,1.62) | (0.84,1.67) |
| *Recent loss of siblings* | 1.09 | 1.19 | 1.07 | 0.98 |
|  | (0.87,1.38) | (0.79,1.80) | (0.77,1.47) | (0.75,1.28) |
| *Distant loss of siblings* | 1.00 | 1.27 | 1.19 | 1.04 |
|  | (0.84,1.18) | (0.93,1.72) | (0.94,1.50) | (0.86,1.26) |
| ***Lack of social contacts*** |  |  |  |  |
| Live alone | 0.97 | 0.69 | 0.37** | 0.58* |
|  | (0.66,1.44) | (0.39,1.25) | (0.21,0.67) | (0.37,0.91) |
| Had no children visiting | 1.11 | 1.16 | 1.08 | 1.19 |
|  | (0.93,1.32) | (0.91,1.49) | (0.88,1.34) | (0.97,1.46) |
| Had no siblings visiting | 1.06 | 0.98 | 0.86 | 1.24 |
|  | (0.93,1.21) | (0.70,1.37) | (0.70,1.05) | (0.97,1.59) |
| Not participating in social activities | 0.95 | 1.06 | 1.02 | 0.91 |
|  | (0.79,1.14) | (0.79,1.42) | (0.81,1.30) | (0.73,1.13) |
| ***Subjective social isolation*** |  |  |  |  |
| Felt lonely | 1.16* | 1.28* | 1.23* | 1.25* |
|  | (1.00,1.33) | (1.04,1.57) | (1.03,1.47) | (1.05,1.49) |
| Had no one to talk | 1.84*** | 5.24 *** | 1.82** | 2.09** |
|  | (1.31,2.59) | (3.57,7.70) | (1.28,2.58) | (1.33,3.28) |
| Had no one to seek for help | 1.05 | 1.40 | 1.06 | 0.91 |
|  | (0.84,1.30) | (0.99,1.98) | (0.80,1.40) | (0.69,1.21) |
| ***Baseline health status(all measured in 2011)*** |  |  |  |  |
| Poor SRH | 2.50*** |  |  |  |
|  | (2.22,2.81) |  |  |  |
| Cognitive impairment |  | 5.50*** |  |  |
|  |  | (4.44,6.81) |  |  |
| Difficulty with ADLs |  |  | 6.67*** |  |
|  |  |  | (5.54,8.03) |  |
| Difficulty with IADLs |  |  |  | 3.77*** |
|  |  |  |  | (3.25,4.38) |
| ***Covariates (all measured in 2011)*** |  |  |  |  |
| Age | 1.01*** | 1.09*** | 1.06*** | 1.10*** |
|  | (1.01,1.02) | (1.08,1.10) | (1.05,1.07) | (1.09,1.11) |
| Male | 0.95 | 0.74** | 0.95 | 0.59*** |
|  | (0.84,1.08) | (0.62,0.89) | (0.82,1.10) | (0.51,0.69) |
| SES | 0.71*** | 0.80** | 1.18** | 0.95 |
|  | (0.64,0.79) | (0.70,0.91) | (1.06,1.32) | (0.84,1.07) |
| Social services | 1.04 | 1.17* | 1.04 | 1.31*** |
|  | (0.93,1.16) | (1.00,1.35) | (0.92,1.18) | (1.14,1.49) |
| Education | 1.01 | 1.01 | 1.02* | 1.00 |
|  | (0.99,1.02) | (0.99,1.03) | (1.00,1.04) | (0.99,1.02) |
| Rural | 0.95 | 0.71*** | 0.71*** | 0.83** |
|  | (0.85,1.06) | (0.61,0.83) | (0.63,0.81) | (0.73,0.95) |
| Smoke | 0.89 | 0.83 | 1.10 | 1.02 |
|  | (0.75,1.06) | (0.65,1.06) | (0.90,1.36) | (0.83,1.24) |
| Drink | 1.41*** | 1.39** | 1.22* | 1.28** |
|  | (1.22,1.63) | (1.12,1.74) | (1.02,1.45) | (1.08,1.52) |
| Leisure | 0.96*** | 0.92*** | 0.96** | 0.96** |
|  | (0.94,0.98) | (0.90,0.95) | (0.94,0.98) | (0.94,0.98) |
| Physical exercise | 0.99 | 0.94 | 1.06 | 0.84 |
|  | (0.85,1.16) | (0.75,1.18) | (0.88,1.27) | (0.70,1.00) |

Note: Multiple-imputation estimates, imputations=10

* *P* <0.05, ** *P* <0.01, *** *P* <0.001
